# Impact of positive fluid balance on survival in critically ill cancer patients

**DOI:** 10.1186/cc10179

**Published:** 2011-06-22

**Authors:** JP Almeida, H Palomba, L Hajjar, V Torres, F Galas, FA Duarte, D Nagaoka, RE Nakamura, J Fukushima, L Yu

**Affiliations:** 1 ICESP, São Paulo - SP, Brazil

## Introduction

Fluid overload has recently been linked to adverse outcomes in critically ill patients, but its impact on the outcomes of cancer patients admitted to intensive care units (ICUs) has not been previously described.

## Methods

A total of 234 cancer patients admitted to the medical ICU in a 6-month period were prospectively evaluated for survival. Univariate and multivariate analyses were used to study ICU admission parameters associated with ICU mortality. Exclusion criteria were ICU stay <24 hours and chronic renal failure on dialysis.

## Results

Overall mortality was 21%. The mean age of all patients was 62.7 ± 11.6 years and 55% were male. Postoperative care (45%) and sepsis (35%) were the major reasons for admission to the ICU. The mean APACHE II score value at 24-hour ICU was 21.2 ± 6.4 and the mean Karnofsky score before ICU admission was 75.2 ± 17.2. At multivariate analysis, the following variables at ICU admission were significantly associated with ICU mortality in cancer patients: Lung Injury Score >2 (OR = 3.3; 95% CI = 1.32 to 8.23) and positive fluid balance (for each 100 ml/24 hours) (OR = 1.03; 95% CI = 1.01 to 1.06).

## Conclusions

Fluid overload is independently associated with increased mortality in critically ill cancer patients. Further studies are necessary to determine the impact of positive fluid balance on acute organ dysfunction and overall prognosis of cancer patients.

